# Domiciliary dental care: a scoping review of the literature

**DOI:** 10.1038/s41405-026-00451-y

**Published:** 2026-06-12

**Authors:** Megan Roberts, Rakhee Patel, Rebecca Ruth Wassall

**Affiliations:** 1 Swansea Bay University Health Board, NHS Wales, Swansea, UK; 2 Faculty of Dentistry, Oral & Craniofacial Sciences, King’s College London, London, UK; 3 School of Dental Sciences, Faculty of Medical Sciences, Newcastle University, Newcastle upon Tyne, UK

**Keywords:** Gerodontics, Special care dentistry, Dental clinical teaching, Dental post graduate education, Dental public health

## Abstract

**Aim:**

Domiciliary dental care is the provision of dental care for those patients who are unable to attend a clinic due to a disability or impairment. This scoping review aimed to identify developments in the literature regarding the provision of adult domiciliary dental care.

**Materials and methods:**

The Arksey and O’Malley framework was used and findings were reported in line with PRISMA-ScR. Subject specific library support was used to produce a search strategy using MeSH (Medical Subject Headings) terms and key words to identify studies published in English between January 2009 and October 2025 relevant to the topic area. Database searching of Medline, Embase, PsycINFO and CINAHL was conducted alongside hand searching.

**Results:**

In total, 45 primary research papers were included. Study types included qualitative research, observational studies and economic evaluations. Key themes identified included patient and staff reported barriers, facilitators to enable or improve domiciliary dental care and the role of dental team skill mix and education.

**Discussion:**

There is currently an absence of rigorous research into dental care in domiciliary settings, likely due to ethical, financial and logistical complexity of undertaking research in this area. Research that has currently been undertaken is mainly focused on older patients living in care facilities.

**Conclusions:**

Domiciliary dental care is an essential service for patients unable to attend a dental clinic due to a significant disability or impairment. Whilst a formal quality assessment of individual studies is not carried out in scoping review methodology, the results of this review identified a number of barriers to its provision. Appropriate risk assessment, education and the effective use of technology were identified as possible facilitators. Further research is needed to enable the provision of evidence-based care to this vulnerable population.

## Introduction

Domiciliary Dental Care (DDC) can be described as a service that reaches out to those who cannot reach a service within a clinic setting. There are various key elements to this provision, including referral triage, an assessment process, person centred care planning and the delivery of the agreed dental treatment at the most appropriate location.

Within this article, DDC is considered to be all aspects of dental care that is provided to a patient within the environment where the patient is currently residing, as opposed to the care that is delivered in dental clinical settings. It includes patient’s own homes, care homes, and hospital wards. Whilst it will include preventative care, this article has not considered dental screening procedures for epidemiological research or public health purposes.

Within the UK, DDC is largely considered a part of special care dentistry, allowing delivery of care for adults who have a physical, sensory, intellectual, mental, medical, emotional, or social impairment or disability or a combination of these factors. It is important to note that the provision of special care dentistry is provided across primary, community and hospital settings [1]. DDC is an example of a reasonable adjustment that may improve access to dental services for some patients with disabilities [2].

DDC is often thought of as a service for older patients experiencing frailty but may also include younger patients for whom the clinical environment may exacerbate ‘challenging behaviour’. The need for DDC will continue to rise with people living longer with increasingly complex co-morbidities whilst also retaining teeth [3]. A survey by Public Health England undertaken of adults in contact with domiciliary dental care services across England found that, when compared to the findings of the 2009 Adult Dental Health Survey, those requiring DDC experienced higher levels of pain and less frequent daily mouth care [4]. However, access to DDC for those that need it is difficult, with a lack of services and significant variations across the country. For those delivering DDC, a standard operating model does not exist which adds another layer of heterogeneity in the services offered to patients requiring DDC.

The aim of this review was to identify developments in the literature in recent years regarding the provision of DDC for adult patients. A scoping review was felt appropriate to enable a broad exploration of the topic and identify any gaps in the evidence. The findings of this literature review have informed the development of new domiciliary dental care guidelines that are currently being produced by the British Society of Gerodontology (BSG) and the British Society of Special Care Dentistry (BSSCD).

## Materials and methods

**Fig. 1:**
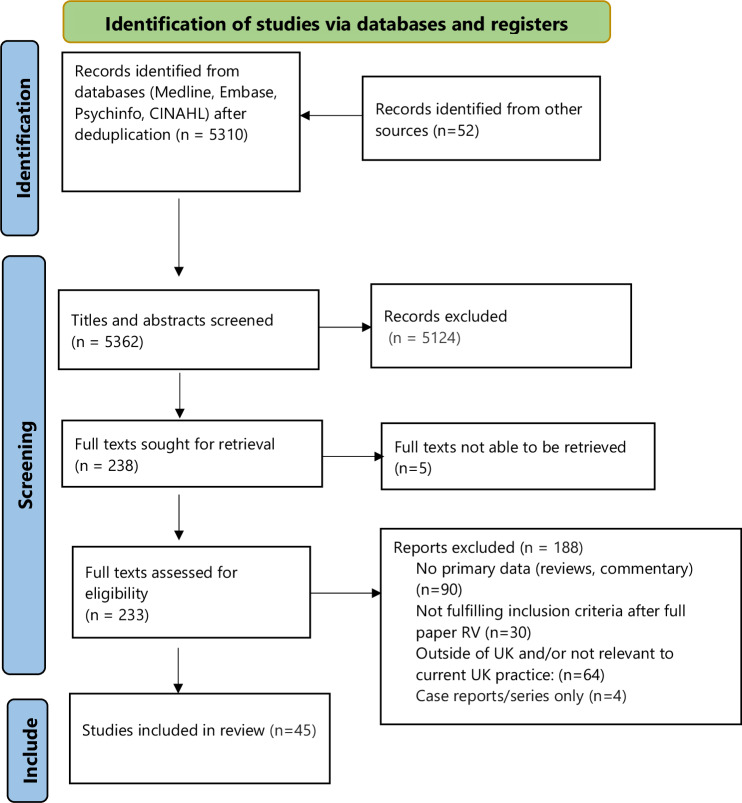
PRISMA flowchart detailing search and study selection process.

We conducted this scoping review using the Arksey and O’Malley framework [5], with a search question identified as ‘When and how should DDC be delivered for adult patients?’. A protocol and data screening form were developed to aid this process. The protocol was not registered a priori. The results were reported according to the Preferred Reporting Items for Systematic Reviews and Meta-Analyses extension for Scoping Reviews (PRISMA-P) as shown in Fig. 1 [6].

### Search strategy and selection criteria

A medical librarian supported the development of a broad search strategy focused around the provision and delivery of DDC. A broad search was undertaken to ensure the maximum number of relevant manuscripts were detected. Four electronic databases were searched for a 16-year period from 1st Jan 2009 to 27th October 2025: MEDLINE, CINAHL, Embase and PsycINFO.

An example of the search strategy that was developed for the MEDLINE database utilising keywords and Medical Subject Headings (MeSH) terms can be found in Table 1. These were grouped under two main concepts including oral health, and the various domiciliary settings. Boolean operators (“AND”, “OR”) and truncations were used to combine and expand search terms. This was then repeated for the other databases. Additionally, the reference lists of papers satisfying the inclusion criteria were manually searched for additional relevant studies, as well as relevant speciality journals that were not included in the four databases.

**Table 1:** Example search strategy.

Ovid MEDLINE(R) <1946 to October week 2 2025>	
1.exp Dental Care/	36,271
2. exp dentistry/	45,6739
3. exp oral health/	23,379
4. (oral health care or oral healthcare).tw,kf.	5649
5. (dental or dentist*).tw,kf.	300,749
6. 1 or 2 or 3 or 4 or 5	595,335
7. exp Home Care Services/	53,470
8. exp Residential Facilities/	61,439
9. exp Homes for the Aged/	15,581
10. exp Nursing Homes/	47,060
11. exp Long-Term Care/	29,893
12. exp Institutionalization/	8934
13. domiciliary.tw,kf.	2755
14. (home care or homebound or housebound).tw,kf.	23,546
15. (residential home* or residential care or long stay hospital* or hospitali#ed).tw,kf.	153,214
16. institutionali*.tw,kf.	17,571
17. long term care.tw,kf.	27,066
18. care home*.tw,kf.	5411
19. 7 or 8 or 9 or 10 or 11 or 12 or 13 or 14 or 15 or 16 or 17 or 18	319,151
20. exp Prisons/ or exp Schools/	174,221
21. 19 not 20	317,818
22. 6 and 21	4070
23. limit 22 to dt=20090101-20251017	2102
24. limit 23 to english language	1969

Medline.

The decision was made to limit the search to studies published since 1st January 2009, which was when the last national domiciliary guidance was produced within the UK [7].

### Study screening

All results were imported into EndNote for deduplication followed by subsequent screening. A single reviewer conducted title and abstract screening of all retrieved citations against the inclusion criteria (MR). A data screening form developed by MR and RW was used to aid this process. As a second reviewer, RW screened a sample of the retrieved citations and these were discussed with MR part of an initial training and calibration process.

### Eligibility criteria

Inclusion and exclusion criteria were developed by all authors to focus on the provision and delivery of domiciliary dental care for adults. Only studies from North America, Australasia, Western and Northern Europe were included, as these regions were considered similar both economically and in terms of the intervention of interest. Studies needed to be in the English language. Eligibility criteria are detailed in Table 2.

**Table 2:** Inclusion and exclusion criteria.

Inclusion criteria	Exclusion criteria
Related to the provision and delivery of DDC for adults (16+ years)	Studies only focused on the epidemiology or oral health status of those receiving DDC Dental care provided on mobile dental units
Teaching and education for dentists and dental care professionals about DDC	Teaching and education for non- dental professionals, including oral health improvement programmes not involving dental staff
English language	Non-English language
Published since 1st January 2009	Published before 1st January 2009
Studies from North America, Australasia and Western and Northern Europe and relevant to the provision of care in the United Kingdom	Related to care in other parts of the world, or lacked relevance to practice in the United Kingdom
Containing primary data (qualitative or quantitative) but excluding case reports	Related to daily oral mouthcare provision only, or non-dental/oral health domiciliary care

### Data extraction and reporting

MR captured the study using data extraction tables (see online supplementary information), including type and geographical location of the evidence, numbers included in the study (where relevant) and a summary of key findings. This was checked by the 2^nd^ reviewer (RW) for accuracy.

Two reviewers (MR & RW) worked together to extract key findings from the included articles. Next, data were thematically organised into barriers and facilitators to the provision of DDC. This was an iterative process that was undertaken with the aim to describe key concepts and identify gaps rather than synthesising evidence. Lastly, a narrative account was developed by the authors, drawing on their clinical experience of providing DDC, to interpret the findings, identify connections between themes and draw conclusions where possible.

### Ethics

Ethical approval was not relevant for this literature scoping review, which used only secondary data. The authors undertook this work as part of domiciliary dental care guideline development which is a joint venture between the British Society of Gerodontology and the British Society of Special Care Dentistry. RW is the immediate past-president of the British Society of Gerodontology but this work is not financially supported by either organisation.

## Results

The search for primary research studies generated 5310 unique citations with 52 citations from additional sources. The full-text of 238 studies were retrieved as potentially relevant, of which 45 studies satisfied the inclusion criteria. The reasons for excluding studies are detailed in Fig. 1 alongside the retrieval, screening and selection processes.

### Characteristics of included studies

Included studies were largely qualitative in nature (*n* = 23), frequently exploring attitudes and experiences of patients, carers and dental care professionals regarding DDC. The numbers of participants in qualitative studies ranged from 9 to 177. Other types of studies included a wide range of methodologies such as economic evaluations, observational studies, correlational study and review of surveys.

**Fig. 2:**
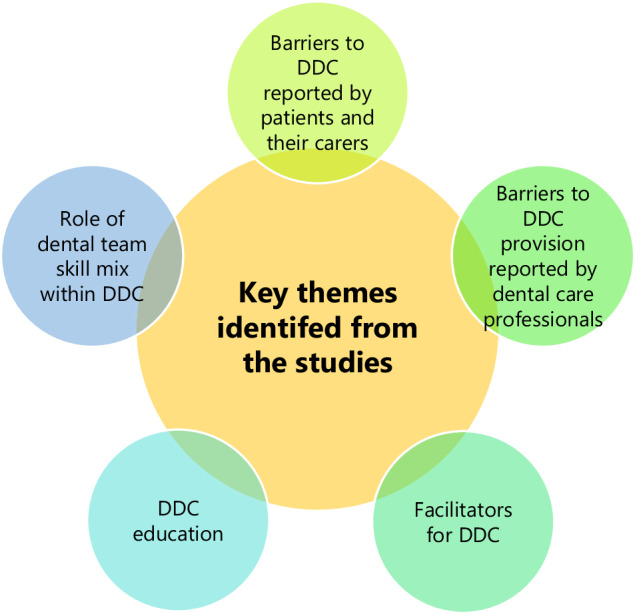
Key themes identified from the studies.

Most of the included evidence was from the United Kingdom (*n* = 15), with the rest from Europe (*n* = 13), North America (*n* = 11) and Australasia (*n* = 6). The key themes identified in the studies are outlined in Fig. 2.

#### Barriers for domiciliary dental care

The barriers faced when seeking and delivering DDC have been well reported in the UK and other countries, and the qualitative literature includes experiences of patients and their care providers [8–14], dentists [8, 13, 15–20], and dental care professionals [21, 22].

##### Barriers for patients and carers

Access to routine DDC services was recognised as important, although can be difficult [8–10]. Geographic variations exist that do not always correlate to levels of need [14]. A lack of awareness about existing service provision was reported to be a barrier to accessing care [9, 12], as well as financial constraints [8, 11]. An examination of 848 English care home residents found 40% to require domiciliary services if dental care was needed, commonly due to medical complexities, significant mobility issues or severe confusion and distress [23].

##### Barriers for dental professional

Clinician’s concerns about inadequate remuneration for DDC was a common theme in the literature and was often cited as a reason for their hesitance to offer regular DDC, both in the UK and internationally [15, 16, 18–21]. Other consistently reported issues include treatment complexity, especially with many patients retaining heavily restored dentitions later in life [16, 18]. Medical complexities, or uncertainty regarding patient medical histories [8, 16, 21], patient social complexities [21], and concerns regarding consent and capacity were also stated as barriers to care [19, 22]. The issue of patient payment for DDC treatment was identified as problematic, especially when patients lack capacity and when those caring for them are uncertain of their financial status [9]. Dentists report being apprehensive about litigation [16], the inability to provide high quality care on a domiciliary basis [18], and patient and staff safety [17, 18]. A lack of training and experience and desire for further training was commonly noted [15, 21, 22]. A small comparability study of clinic-based and home-based oral examinations of older people found that the estimates of oral disease prevalence were generally similar, but there was evidence of systematic bias with home-based examinations detecting fewer carious lesions, restorations and bleeding on probing than the clinic-based examinations [24].

#### Facilitators for domiciliary dental care

Both clinicians, patients and their carers recognised DDC as an important service to provide holistic, patient centred care [21, 25]. Clinicians who provide DDC generally report personal and professional satisfaction and development [13, 15, 21], but this was not universal [20].

##### The role of technology

In recent years, use of tele-dentistry as an adjunct or alternative to DDC has been described, although mainly outside of the UK. The term tele-dentistry is broad, varying from virtual assessments to the use of intra-oral technologies. In Germany and the USA, studies involving tele-dentistry examinations within care home settings using intra-oral scanners compared with traditional face to face examinations have been explored [26, 27], although accuracy discrepancies were found when assessing caries or plaque levels compared to examinations regarding missing teeth or the presence of restorations [27]. Another tele-dentistry study using endoscopy equipment and lighting compared with face-to-face examination on the same patient found tele-dentistry examinations were quicker and showed high levels of sensitivity and specificity compared with conventional examinations [28]. A further study conducted in the USA using mobile radiographs and photographs found concordance between asynchronous tele-dentistry and in person examinations was substantial for surgery and removable denture treatment decisions and moderate for restorative needs for care home residents [29].

One New Zealand study found three quarters of residents in an older person care home thought tele-dentistry was beneficial, with three fifths comfortable having a tele-dentistry consultation or advice, although acceptance was lower amongst the oldest residents [30].

There were two studies regarding the use of handheld radiographic equipment in a care home domiciliary setting, although both involved relatively low numbers of radiographs [31, 32]. 80% of radiographs were of acceptable quality in one study [32], with positioning errors of 10% in another [31], although this was attributed to the complexity of the patients seen. Staff dosimetry was found to be well within recommended exposure limits [31].

##### Patient safety

There is limited evidence about the incidence of patient safety events during DDC in the UK, but a review of over 218,000 domiciliary treatment sessions undertaken in Sweden suggest patient safety incidents are low (0.3%) [33]. UK based DDC clinicians vary in their current practices for carrying medical emergency equipment, with a survey of 29 DDC clinicians reporting only 1 medical emergency [34].

An e-Delphi study was undertaken involving 24 UK based DDC clinicians to compile a consensus risk assessment proforma with a view to identifying and mitigating patient and environmental risks likely to be encountered on a DDC setting [35].

##### Economic considerations

A Swedish health economic analysis compared the cost of DDC to attendance at a fixed clinic, finding DDC be an estimated 15% lower [36]. However, it is difficult to say whether this could be extrapolated to a UK setting. A Belgian economic evaluation found preventative care combined with care home based curative dental care was more cost effective than preventative care alone or preventative care combined with community clinic care [37].

Marino et al. [38] undertook economic modelling and found, unsurprisingly, that asynchronous tele-dentistry intra oral camera recordings undertaken by a registered nurse was the most cost-effective form of dental assessment and treatment planning, whereas face to face assessment by a dentist was the least cost effective. They suggested that this form of assessment may have a role in improving access for residents in rural long term care facilities who may otherwise not be able to access care.

##### Silver diamine fluoride

Three studies looked at the use of silver diamine fluoride (SDF) for caries prevention and/or treatment [17, 39, 40]. One small trial found a single application of 38% SDF on a domiciliary setting did not have a statistically significant preventative effect on root caries development compared with a preventative programme alone [39]. However, another trial looking at 188 active carious lesions applied SDF on a domiciliary setting to existing caries and found that 83% of teeth showed signs of arresting caries at 3 weeks compared to 0% of lesions in the same patient with no intervention [40]. This technique was agreed to be non-invasive and suitable for placement on a DDC setting by a small group of General Dental Practitioners (GDPs), although knowledge of its use varied [17].

##### Use of dental team skill mix

Effective use of skill mix within the dental team is seen as a key feature for future provision of DDC, and dental care professionals report direct access is likely to have a positive impact upon care for older adults [22]. One UK study found that a significant proportion of the treatment needs of care home residents could be provided by dental hygienists, therapists or clinical dental technicians [41], and that the ability to provide treatment increased further with additional special care experience.

A further paper from this study exploring treatment needs found GDPs could theoretically provide care for half of care home residents, with 40% needing care by a dentist with special care experience and fewer requiring input by a specialist in special care dentistry. Those that needed specialist input had higher number of retained teeth and high interventional treatment needs. It was felt DDC was required forsome or all treatment for half of the care home residents assessed [42].

##### Domiciliary dental education

Studies evaluating DDC education were few in number, and those that were published typically included low numbers of participants. When dentist and dental hygiene students were exposed to clinical DDC experience as undergraduates this was generally received positively, giving students a greater appreciation of the importance of dental care for care home residents [43–45], and transferable communication and adaptability skills [44]. Some studies highlighted the challenges of DDC compared to clinic settings [43, 46, 47], with the provision of scenario-based video training improving feelings of preparedness compared to a control group [47]. The deficiency in dental hygienist DDC education was highlighted [48]. One study showed no impact of a dental undergraduate curriculum on knowledge on ageing or attitude towards institutionalised elderly people as perceived by recently graduated dentists [49], whereas a different study showed a reduction in willingness to treat housebound and frail elderly patients by American dental students as they progressed from the first to fourth year of dental school [50]. The inclusion of dental hygiene students in care conferences for care home residents was found to improve student’s understanding of patient complexity and interdisciplinary communication [51].

No studies included experience or teaching specific to patients receiving DDC in their own home, with most studies referring to institutionalised individuals only.

## Discussion

This scoping review identified 45 primary studies relevant to the provision of domiciliary dental care, mapping topics with particular relevance to UK DDC practice. Key themes included barriers and facilitators for domiciliary dental care, including factors such as education and experience, use or availability of the dental team and practical considerations for providing DDC. Oral conditions can impact the general health, diet [52], and quality of life of older adults [53]. Domiciliary dental care and daily oral care strategies must therefore focus on preventing disease, managing pain, reducing co-morbidity and supporting quality of life.

This scoping review had some limitations. The inclusion criteria excluded articles not written in English which might exclude perspectives from non-English speaking countries. Furthermore, only studies from North America, Australasia and Western and Northern Europe were included, as these regions were considered similar both economically and in terms of the intervention of interest. However, the authors recognise this may have excluded potentially relevant evidence.

Although this study is a scoping review rather than a systematic review which is considered more rigorous, the use of a broad structured search strategy, PRISMA and extensive searching of grey literature from subject relevant publications helped identify relevant research in this specific field.

There is an absence of rigorous research in domiciliary settings. This is likely due to ethics, costs and contextual complexity that make certain research methods more feasible [54]. It is therefore important to weave together different types of studies with other evidence from stakeholders to ensure any resulting guidelines are applicable to real-world practice. It is therefore difficult to make firm conclusions or recommendations in many of the areas covered due to a lack of rigorous research in these areas. One area that was most comprehensively covered in the literature is qualitative research exploring the perspectives of dental care professions and care home staff regarding DDC.

There were notable gaps in the literature. There is a lack of evidence into cost effectiveness, feasibility and acceptance of DDC service provision within the UK. A 2020 study involving practitioners with significant DDC experience expressed the need for further guidance about information governance, appropriate domiciliary equipment and medical emergency equipment requirements [35]. UK guidelines and standards partially address some of these issues, with a focus on risk assessment based on the proposed treatment, patient complexity and DDC location [55, 56].

Although a few studies looked at the roles of different members of the dental team, this is an underexplored area. A cluster randomised controlled trial to determine whether members of the dental team other than dentists could improve the oral health of dentate older adults living in care homes is currently being undertaken, however full findings of this study are not yet published. A study of initial process evaluation findings with key stakeholders corroborated other studies regarding access difficulties and barriers faced by care staff, but suggested openness towards dental hygienist-therapist input in this area [57]. The publication of these results will likely be a valuable addition to the evidence regarding skill mix use.

Although minimally invasive techniques such as silver diamine fluoride (SDF) [17, 39, 40] and atraumatic restorative technique (ART) [58] may be potentially useful for domiciliary dental care, the evidence is limited, setting-specific, and often based on small studies with refuted methodology [59]. Conclusions regarding acceptability, feasibility and clinical efficacy of their use in this setting need further well controlled research before its use can be recommended for DDC settings. There were only two studies on the use of handheld radiography equipment on a domiciliary setting [31, 32], and it was not possible to determine how commonly this equipment is used in the UK or elsewhere. It is recognised that ideal positioning can be problematic in the domiciliary setting and it is essential that local protocols are developed to align with regulatory requirements [60]. Based on the available evidence, it is not possible to determine whether handheld radiography equipment is appropriate for domiciliary settings.

Much of the current evidence overwhelmingly focuses on patients who reside in care facilities rather than individual’s own home. This is likely due to logistical ease in undertaking research where multiple participants may be gathered in one location. However, home dwelling patients who require domiciliary care may have unique characteristics and challenges that are not fully understood. It is essential that further research in these settings is undertaken so that conclusions can be drawn, and guidance created, that is relevant to all people who require DDC.

## Conclusion

This scoping review of published literature about domiciliary dental care identified a number of barriers to its provision and found risk assessments, effective education for the dental team and used of technology were possible facilitators. Whilst a formal quality assessment of individual studies is not carried out in scoping review methodology, the results of this review suggested that, for patients unable to attend a clinic due to disability or impairment domiciliary dental care, is an essential service. It is the author's opinion that the field of DDC requires investment in high quality research to support the development of professional practice and enable the provision of evidence based care to this vulnerable population.

## Supplementary information

Overview of included studies

## Data Availability

Data supporting the findings of this study are available in the supplementary information document.
