# Supplementary material for: Domiciliary dental care: a scoping review of the literature
Source: BDJ Open. 2026 Jun 12;12:61. doi: 10.1038/s41405-026-00451-y (PMC13263353; doi:10.1038/s41405-026-00451-y)
Supplement: Supplementary file 1 — Overview of included studies [file 41405_2026_451_MOESM1_ESM.pdf]

Author, Year Country, Study design	Aim of study	Key outcomes	Participants	Key findings
Emanuel R et al. 2024.<br><br>UK (South England)<br><br>Design: Qualitative-thematic approach	To identify how dental therapists (DT) and dental therapy educators perceive domiciliary dental services. Explore the role and experiences of dental therapists in providing domiciliary dentistry including limitations.	Feelings and experiences	Final year student Dental Therapists (n=7)<br>Qualified Dental Therapists (n=10)<br>Dental Therapy educators (n=9)<br><br>Total n= 26	Barriers: financial (patient and DT), limited training, prescribing barriers, lack of awareness of service by patients, medical and social history factors<br><br>Benefits for DTs: professional development<br>Financial provision into service and workforce support essential<br>Private and NHS DDC providers required to fulfil need
Angst L at al. 2022<br><br>Switzerland<br><br>Design: Qualitative	Attitudes of GDPs on caring for older adults, with focus on Silver Diamine Fluoride (SDF) and DDC	Awareness, experience and feelings	n=17 general dental practitioners (GDPs) working in Switzerland (with and without current DDC experience)	Themes:<br>Varying knowledge of SDF. Varying opinion regarding importance of SDF staining. Agreement on low invasiveness of this treatment. Product availability issues.<br><br>DDC: Positive views on providing DDC. Concerns: lack of infrastructure, remuneration, safety for patients and staff
Mintz, E. et al. 2022<br><br>UK (Sussex, England)<br><br>Design: Qualitative	To explore barriers to providing DDC (and preventative care), to older patients including those with dementia and how this can be overcome	Thoughts and opinions	Semi structured interviews<br>N=15 clinicians<br>NHS+/or Private GDPs (10) and Foundation dentists (5)<br>FDs had no DDC experiences, GDPs no recent experience, and experience limited to mainly emergency visits to care homes	Benefits of DDC: professionalism, clinical satisfaction, holistic care<br><br>Barriers to DDC provision: GDC regulations, contract changes, financial concerns, administration issues, practicalities (equipment/staff), ‘compromised’ care

Kerr E et al. 2021<br><br>Northern Ireland<br><br>Design: Qualitative descriptive Study	GDPs attitudes and barriers to providing DDC	Feelings and experiences	N= 12 GDPs (private and NHS) working in Northern Ireland	Major Themes: risk of litigation, remuneration, complexity of treatment, overall framework of dental care in NI<br><br>Minor themes:<br>Culture within individual practices impacted DDC 1provision. Overall positive attitude towards DDC
Smith, M. &Thompson, WM. 2017<br><br>New Zealand<br><br>Design: Qualitative	Challenges dentists face when providing DDC to older adults and how these challenges could be overcome	Thoughts and feelings	Interviews with n=20 dentists who currently or previously provided DDC	Patient barriers: financial for patient, lack of awareness and access to services, patient disability, 'self-ageism' views on need for dental care<br>Dentist views: Lack of suitable renumeration for dentist, professional obligation and felt DDC important. Concerns about treatment planning and risk of supervised safety. Importance of skill mix and team working. Need for training for dentists and care home staff.
Bots-vantSpijker et al. 2016<br><br>The Netherlands<br><br>Design: Questionnaire	Perceived barriers in providing care for community dwelling frail older people	Thoughts and feelings	N=595 dentists- general	Limited opportunity to refer to colleagues with specialist knowledge and experience<br>Barriers: knowledge, patient cognitive impairment and other practical barriers<br>42% not willing to provide DDC periodic oral examination for housebound patients. Poor reimbursement
Chowdhry, N. et al. 2011<br><br>Canada<br><br>Design: Questionnaire	Compare perceptions of dentists about providing treatment in long term care facilities (LTCF) between 1985 and 2008	Thoughts and feelings	N= 251 dentists- 15% treating in long term care facilities (LTCF), 19% history of treating in LTCF	Considerations of those currently providing:<br>Continuing education, support from dental team, remuneration<br>Benefits: increase number of patients seen, broaden clinical practice<br>Feelings those who stopped providing: financially unrewarding, professionally unsatisfying<br>Compared with 1985: more deterred by admin difficulties and finances.<br>Reduced % with advanced education in geriatrics between 1985 and 98.
Janssens, L et al. 2025<br><br>Belgium	Outlines the feelings of care home managers whose care homes are involved in a	Thoughts and feelings	Interviews with care home managers (n=10) from 6 care homes	Key themes:<br>Managers prefer DDC as felt it overcome transport and access barriers. They are keen for reliable partnerships with oral health professionals. They recognise value of DDC to the care home but feel there are unmet needs.

Design: Qualitative	specific DDC scheme			Financial concerns are the key barrier to implementation of DDC. They felt DDC should adapt to the local context.
Patel R. et al. 2021<br><br>UK (London)<br><br>Design: Qualitative	Present the findings of the challenges relating to access to dental care for older people in care homes from the Fluoride Interventions in Care Homes (FInCH) Trial	Thoughts and experiences related to dental access in care homes	Interview and focus groups with staff (N=22) from 3 care and 3residential homes. Care home managers, carers, 1 nurse.	Availability: issues with routine care, non private care, dentist availability<br><br>Affordability: uncertain exemption status, cost of treatment and transport<br><br>Accommodation: complex referral system, lack of staff to chaperone, waiting times<br><br>Acceptability: oral care not priority, dentist confidence and experience, resident complexity<br><br>Accessibility: lack of transport, lack of clarity about available services, limited service availability
Hearn, L. & Slack-Smith, L. 2016<br><br>Perth, Australia<br><br>Design: Qualitative	To explore elderly care home staff perspectives on how to engage dental professionals in the provision of oral care for residents	Thoughts and feelings	N= 30 staff from 6 facilities (nurses, carers, nurse directors)<br><br>Open interviews	Barriers: financial, dentist unwillingness, waiting times, bureaucracy, transport, knowledge of how to find dentists, lack of knowledge about oral health, dentist inflexibility, lack of dentist knowledge and experience<br><br>Suggestions:<br>Better collaboration, increase frequency of oral health reviews, improved education, use of skill mix.
Komulainen K. et al. 2012<br><br>Finland<br><br>Design: Mixed methods study	Explore factors associated with preference for home visit among home dwelling older people	Thoughts and feelings compared with epidemiological data	N=321 home dwelling older people in Kupio city, Finland	26% expressed a preference for home visits.<br>Those with this preference had higher use of home services, fewer own teeth, high levels of periodontal pocketing and lower use of primary care services.
Monaghan, M & Morgan, M. 2010.<br><br>UK (Wales)<br><br>Design: Questionnaire	Survey of factors that could facilitate or impede access to dental care for care home residents	Experiences and thoughts	Survey of care home managers, with additional interviews<br><br>N= 957 questionnaires, 123 interviews	Issues: access to routine and emergency care, onsite facilities for DDC provision, provision of daily care and associated carer training, assumptions made about ability to eat foods, assessment upon admission to care home.

Makansi N et al. 2021<br><br>Canada<br><br>Design: Qualitative descriptive	Understand how existing DDC services operate to inform development of practice models and provide recommendations	Observations of DDC visits<br><br>Feelings and experiences	Observed 23 DDC visits in long term care facilities (LTCFs) and private home<br>7 semi structured interviews with clinical and non-clinical dental staff involved in DDC, 4 caregivers and 2 patients (n=13)	Dental staff: Motivations to provide DDC: altruism, positive experience, positive impact on personal life.<br><br>Skills required: patience, adaptability.<br>Challenges: emergency visits<br>Remuneration – described both positively and negatively<br><br>Caregivers/patients reported: expressed gratitude and felt eliminated barriers for dental care
Ahmad B et al. 2018<br><br>County Durham, England<br><br>Design: mixed methods design-survey, focus group, interviews	Identify barriers to provision of oral healthcare for older people in care homes, to inform the development of an oral healthcare strategy	Access to policies, dental care and staff training<br>Thoughts and feelings	Care home managers and carers, dentists, (OHI team reps, commissioners, academics + others<br><br>N=93 postal questionnaires, interviews n=28	Disparity between reported oral health care provision by care home managers (positive) vs interviews with non-care home managers (felt oral health needs not adequately met).<br><br>Identified issues: lack of access to routine care. Lack of denture labelling/lost dentures/issues with remaking dentures. Variation in access. Lack of carers' knowledge of medical history and patient's obligation for payment. Poor uptake of free oral health training to care home staff
Caplan et al. 2022<br><br>Iowa, USA<br><br>Study type: Longitudinal study	To assess the relationship between the receipt of dental procedures and mortality among a cohort of nursing facility residents.	Mortality amongst a cohort of older age residents over at least a 5 year period	Residents of 10 care homes (n=535)	A decrease of 13% (dental preventative procedures) and 16% (prosthetic procedures) in hazard of death for each unit increase in the number of intervals during which dentate residents underwent at least one procedure, after controlling for age, sex, and statistically significant health conditions. Among edentulous residents, prosthetic procedures were not significantly associated with survival. No evaluation of causality was undertaken.
Werbrouck A at al. 2022<br><br>Belgium<br><br>Design: Economic evaluation	A health-economic evaluation of oral healthcare delivery in institutionalised older people	Incremental cost effectiveness ratios and healthy oral years	Scenario and sensitivity analysis<br>Financial operational costs and disease costs	Comparison of 1) usual care 2) on site preventative care only based on recommended guidance 3) preventative care as option 2 with community based curative dental care 4) preventative care as option 2 with on-site curative dental care.<br><br>Preventative care alone and preventative care with community curative care not cost effective compared to preventative care combined with on-site

				curative dental care. Preventative care with on-site curative care over 10-year period would cost €2628 per nursing home space for this approach compared to usual care with anticipated additional 2 'healthy oral years' but further evaluation required.
Geddis-Regan A. & O'Conner R. 2018<br><br>UK<br><br>Design: Correlational study	Assess NHS payment claims for DDC to look for variation by area, age and level of deprivation	Service provision of NHS DDC	NHS business service authority data in England in 2015 + CCG data regarding demographic of population by area (proportion of older adults, level of deprivation)	Substantial variation by area in the number of NHS DDC claims.<br>A statistically significant but very weak positive correlation between population of each area and the number of DDC claims.<br>Positive but weak association between area deprivation measures and the number of claims per population.<br>There was little evidence of an association between proportions of older adults and numbers of DDC claims
Sjögren et al. 2015<br><br>Sweden<br><br>Design: Retrospective observational	Assess patient safety during DDC	Number of safety events during DDC	218,586 DDC treatment sessions for elderly nursing home residents over a 2 year period.<br>39110 patients. Mean age 86.<br>Included assessments (80%) and treatment (20%)	Patient safety events (0.03%) low<br>Most common serious safety events were patient identity control related
Tomson M. et al 2015<br><br>UK (West Midlands)<br><br>Design: Epidemiological survey	Determine the oral health status of care home residents and provide data to inform local needs assessments and commissioning decisions.	Epidemiological survey	N=848 care home residents examined and patient reported questionnaire	Just under 40% of participants in the clinical examination were reported by examiners to require domiciliary services if care was needed. Common reasons given included 'severe medical problems', 'significant difficulties with mobility' and 'very confused and distressed'.
Fairhall et al. 2009<br><br>New Zealand	Determine whether home-based and clinic-based dental examinations of	Accuracy of dental examination (missing, filled and carious teeth,	Dentate patients (n=61) age 66-74 living in their own homes	Comparability of clinic-based and home-based oral epidemiological examinations for older people. It has found that the estimates of oral disease prevalence were generally close , but evidence of systematic bias with home-

Type of study: Comparative study	dentate older people are comparable	clinical attachment loss)		based examinations detected fewer carious lesions (average 0.5 teeth), restorations and bleeding on probing than the clinic-based examinations
Geibel, A.M. et al. 2025<br><br>Germany<br><br>Study type: Observational	Assess quality, and diagnostic use for treatment planning of radiographs taken using Nomad Pro2	Diagnostic quality of intraoral radiographs	Single tooth radiographs (n=127) taken in a retirement or nursing home setting by a single experienced operator	80% showed no issue with quality of radiograph, 4% of no diagnostic use. This is higher % than in normal surgery environments. Sufficient field of view for 84%.
Kharbot, B. et al. 2025<br><br>Germany<br><br>Study type: Proof of concept study	Telemedical examination of the dental status of functionally dependent older people residing in care facilities.	Accuracy and reliability of intra-oral scan reviews compared with traditional examinations	Residents age 65-95 at 4 residential facilities (n=63)	Comparison between traditional examination (missing teeth, restorations, cavitated caries lesions and plaque levels) and examination from review of images taken using intraoral scanner. Accuracy for detecting missing teeth or restorations was higher than detecting caries or plaque levels. Intra-rater reliability was very good for detecting restorations and good for caries.
Castelaz, M. et al. 2025<br><br>Iowa, USA<br><br>Study type: Qualitative	To explore experiences with a 'virtual dental home' among community dental clinic long term care facility staff	Thoughts and feelings	N=9 staff members: dental staff, long term care facility staff including managers and clinical director	Reviews the virtual dental home (VDH) concept- piloted this model of asynchronous tele-dentistry that enables patients to connect with a 'dental home' (eg clinic) and receive diagnostic and preventive services in community-based settings.<br>Dental hygienists saw patients in community-based settings using portable dental equipment and share diagnostic information (e.g., radiographs, intraoral photos, clinical measurements) with a dentist who then conducts exams virtually and determine treatment needs.<br><br>Interview outcomes: felt reduced transport barriers to clinic. However, high administrative burden with greater on dental care team assistance than anticipated. No major technology issues were encountered.
Ng, B.M.M. et al. 2024	Residents (age 65+) and staff of care facilities thoughts	Thoughts and feelings	Residents (n=100) of 14 rest home level care facilities and staff	3/4 of residents thought that teledentistry was beneficial and 3/5 residents were comfortable receiving remote dental consultations and advice.

New Zealand<br><br>Study type: Qualitative	regarding acceptability of tele-dentistry		(n=77)	Acceptability was lower among older participants. Staff participants were receptive to teledentistry use for residents.<br><br>Residents perceived benefits: convenience, timesaving, energy-saving, reduced time on a waiting list, reduced pressure on care staff, reduced need to involve family, reduced cost and travel expenses, reduced difficulty in accessing services,<br>Reasons for resident's lack of acceptability: no perceived need, difficulty with new technologies, self-ageism, preference for human interactions and in-person consultations, hearing difficulties
Kandala K et al. 2024.<br><br>North Carolina, USA<br><br>Type of study: Observational	Comparison of initial dental treatment decisions between in-person and asynchronous teledentistry examinations for people in care homes	treatment decision concordance between traditional in-person examinations and asynchronous teledentistry technology	Patients age 18+ living in care facilities (N=100) with special health needs. 81/100 age 65+.	Examination by hygienist including history, charting and mobile radiographs and photographs.<br>Concordance was substantial for surgery and removable denture treatment decisions and moderate for restorative needs. Patient characteristics and facility type were not significant factors in the levels of examiner agreement
Clark R. C et al. 2021<br><br>UK (West Midlands, England)<br><br>Design: Service evaluation, observational	Insight into current practice regarding DDC risk assessments and medical emergency kit		N=29 (11 General dental service, 17 Community dental service) with a clinical role in DDC	Sources of DDC referrals: self-referral, care homes, general medical practitioners, district nurses, other.<br><br>DDC kit outlined- varying availability of straight/contra angled motor, light sources, suction.<br><br>N=1 experienced medical emergency during DDC<br>64% always have emergency drug kit, 21% when providing treatment only, 15% carry no emergency drug kit.<br><br>73% carry out risk assessment. Most have DDC protocol. Significant number (n=10) had received no manual handling training.<br><br>Recommendations made regarding risk assessment to include patient, treatment and environmental factors.

Zaidman,B. et al. 2020<br><br>England and Wales<br><br>Design: Consensus statement	To develop a standardised domiciliary dentistry risk assessment form based on expert opinion	Development of risk assessment tool	N=29 (24) dentists with DDC experience contributed to e-delphi study	Consensus gained on a four page risk assessment form<br>Good consensus on hazards, use of red/amber/green rating and recommended action table<br><br>Regarding developing national DDC guidance, suggested that national policy should include information governance and recommendations regarding equipment and drugs.<br>They concluded partial support for existing BSDH 2009 DDC<br><br>Advised training and piloting of reviewed risk assessment form required before national introduction.
Queyroux, A., et al. 2017<br><br>France and Germany<br><br>Type of study: Observational study	Assess accuracy of tele-dentistry dental examinations compared with face to face examinations	Amount of dental pathology	235 elderly residents across 8 nursing homes In France and Germany	Patients examined twice within 7 days- one by video recording with specialised endoscopy equipment and lighting with dentist located remotely and then face to face by the same dentist in the care home. Patients therefore acted as their own control.<br>The sensitivity of teledentistry for diagnosing dental pathology was 93.8% and the specificity was 94.2%. (4.8%) false positives. The teledentistry assessments were quicker than the face-to-to-face examinations (12 and 20 minutes, respectively)
Jogezi et al. 2016<br><br>UK (England)<br><br><br>Type of study: Mixed methods	Describes the introduction of mobile xray unit into a DDC service and test radiation safety and image quality	Service provision	Safety: 3 month dosimetry of staff (20 patients, 34 radiographs)<br><br>Imagine quality:32 exposures using mobile unit, 32 exposures using fixed unit (n=64) on test	Dose measurements of staff within well recommended levels (<0.01mSV, min measurable dose) based on maximum of 100 exposures/wk<br><br>Lower quality ratings of radiographs taken using mobile vs fixed unit but not quite statistically significant<br><br>Of exposures taken of actual patients, patient positioning errors >10%, but undertaken on special care population so may be expected.
Lundqvist et al. 2015<br><br>Sweden<br><br>Design :Economic study	Analyse economic consequences of providing domiciliary dental care for elderly nursing home residents in	Health economic analysis	Interviews with nursing home staff (n=5), county council officials (n=2) and academic experts in geriatric dentistry (n=3)	DDC estimated cost 15% lower than fixed clinic visit, this was considered cost effective

	Sweden, compared to dentistry at a fixed clinic			
Marino, R., Tonmukayakul, U., Manton, D., Stranieri, A. & Clarke, K. 2015<br><br>Victoria, Australia<br><br>Type of study: Cost analysis	Compare cost effectiveness of implementing and operating tele-dentistry examination (real time or asynchronous) compared to face to face examination	Hypothetical cost of examination and treatment plan development for 100 residents in a residential aged care facility	n/a	Modelling data based on asynchronous intra oral camera recording undertaken by a registered nurse was the most cost effective form of dental assessment and treatment planning, whereas face to face assessment by a dentist was the least cost effective.<br><br>Rationale was explore whether this could be used to improve access for residents in rural long term care facilities who may otherwise not be able to access care.
Turner S. et al. 2020<br><br>UK (Scotland)<br><br>Design: questionnaire survey	Explore challenged to DDC delivery by dental hygienists and therapists	Thoughts, experiences and current service provision	Online survey of all dental hygienists and therapists in Scotland N=196<br>75% worked in GDS (NHS or private)<br>27% provided DDC	Estimated 2/3 of their workload with adults aged 65+<br><br>Specifically related to DDC:<br>Those providing DDC mainly worked in PDS<br>Most common DDC procedures: OHI, S+P<br>Barriers for DDC:<br>Poor hygiene in home, communication, lack of suitable space and equipment, determining capacity, identifying NOK, lack of support care staff, poor denture maintenance, denture cleanliness, insufficient training, insufficient time, multimorbidity, determining exempt status<br><br>Those with experience of direct access (for working with older patients in general, not just DDC) had a positive effect = yes (20%), no effect (27%). 39% did not work on direct access basis
Morgan M et al. 2016	Report the observations of clinical examiners	Epidemiological survey data and	N= 708 care home residents (age 39-102, mean age 85) across	GDP could provide care for approx. 50% of patients (50:50 of these on DDC: primary care basis)

UK (Wales)<br><br>Design: Observational	on the relationship between treatment plans, complexity anticipated in delivering those plans, and the necessary SCD skills/settings	demographics data	213 care homes across Wales	40% needed dentist with special care experience. Less needed SCD specialist- those with high number of retained teeth, high interventional treatment needs,<br><br>92% needed 1 setting for care.<br>DDC needed for some/all of treatment for half of patients
Monaghan N. and Morgan M. 2015<br><br>Wales<br><br>Design: Retrospective observational	Estimate the proportion of care home residents' dental treatment needs which could be delivered wholly by hygienists or therapists	DDC treatment that could be provided by DCPs	5 residents from each of 228 care homes over 22 local authority areas (n=655)	Care home resident treatment could be provided by dental hygienists (22%) or therapists (27%). With special care experience, this could increase to 43%/53%.<br>Dentists with SCD experience could provide 90% of proposed treatment, dentists with no SCD experience could provide 39%<br>Extended duty dental nurses could solely treat 0%<br>Clinical dental technicians cold treat 6% (rising to 12% with SCD experience)
Ericson, D. et al.<br><br>Sweden<br><br>Study type: randomised, single-blinded, placebo-controlled trial	Evaluate additive effect for prevention and treatment of root caries when combined with preventative programme	Presence of caries on root surface	Adults age 70+ seen on a domiciliary basis in a care home with at least 1 exposed root surface (n=356).<br>Study (SDF) group n= 174, placebo group n=182.	A single 38% SDF application to complement a risk-based preventive programme including fluoride varnish applications did not have a statistically significant additional preventive effect on root caries development when assessed after 12 months.
Shakir, H. et al. 2023<br><br>Texas, USA<br><br>Study type: Clinical trial	Effectiveness of a single application of 38% SDF compared with no treatment in arresting and controlling active, accessible surface lesions in adults (18+) living in nursing homes and	Caries status of treated lesions after 3 weeks.	39 adults aged 18 or older with 188 active lesions from nine San Antonio, Texas, nursing home facilities.<br>Treatment teeth (n= 94) and control (n=94, same patient). Mean age 63 (+/- 15 years).<br>50% cognitively impaired.	One drop of 38% SDF solution applied to treated tooth and reviewed after 3 weeks to assess for colour and texture changes suggestive of caries activity (active or arrested). Caries arrested in 82% of teeth in the treatment group, compared to 0% in the control group.

	long-term care facilities			
Beaton et al. 2020.<br><br>Scotland, UK<br><br>Study type: Qualitative	Views and experiences of recent graduates delivering oral healthcare in a care home environment	Views and experiences	Vocational dental practitioners- in first year since graduation (n=10)	A pilot project to explore experienced of exposing recent graduates to the 'caring for smiles' oral health improvement programme in Scottish care homes. They attended care homes with caring for smiles teams for 2 days carrying out oral health assessments and delivering preventative treatments to residents.<br>Key themes: saw dentists role as key within a care home. Increased awareness of challenges in delivering the programme (funding, paperwork, patient management). Greater awareness of oral health status of care home residents and how general health affected provision of oral care. Overall enjoyable and beneficial experience.
Szabo, K.B et al. 2018<br><br>USA<br><br>Study type: Qualitative	Explore dentist hygienists' perspective on how their undergraduate education prepared them for treating older adults in alternative settings	Thoughts and experiences	Qualified dental hygienists currently working with older adults in alternative settings such as care homes (n=15)	Most reported education had not prepared them for working in these settings and that most learning had been once started working in this setting. Described the emotional and physical toll of working with this patient group. Described different treatment goals in this setting compared to conventional clinics. They felt hands on clinical experience at undergraduate level would ease transition to working in this setting.
Yoon and Compton. 2017<br><br>Canada<br><br>Study type: Qualitative	Investigate the dimensions of professional competence that are developed through a placement for dental hygiene students in older adult long term care settings	Thoughts and feelings	Final year dental hygiene students: (n=9 of which 5 participated in focus groups and all completed reflective journals and pre-placement questionnaires)	Students felt the placement gave them a greater understanding of this patient population. They felt they developed transferrable communication skills. They found the transition from clinic to care facility challenging but felt it improved their transferable problem solving and adaptability skills. They gained a greater appreciation of care staff and the importance of interprofessional working.
Major, N eta l. 2016<br><br>Iowa, USA	Assess changes in a group of undergraduate students about treating 13 different	Feelings and willingness to treat	1st-4th year students over a 6 year period (n=132)	The results showed that changes in students' feelings about treating and willingness to treat underserved populations were population-specific rather than universal. Compared to the 1st year of study, the students in later years anticipated feeling more negatively towards treating frail elderly and homebound patients and less willing to treat. The students also became less

Study type: longitudinal study	vulnerable groups, including housebound patients			likely to strongly agree that it is their responsibility as dentists to treat underserved populations as they progressed through school.
Huynh, J et al. 2016.<br><br>British Columbia, Canada<br><br>Study type: Qualitative	Whether the inclusion of undergraduate dental hygiene students at care conference meetings were felt to impacted the awareness of oral health issues and the hygiene students' provision of care and ability to work in a team.	Thoughts and experiences	4th year dental hygiene students (n=8) and healthcare professionals (n=9)	Interdisciplinary care conferences are a regulatory requirement in British Columbia that are held yearly where healthcare professionals, the resident and their family meet to discuss the overall care and well-being of the resident.<br>This study looked at whether the inclusion of undergraduate dental hygiene students at these meetings impacted the awareness of oral health issues and the hygiene students' provision of care and ability to work in a team.<br><br>Key outcome themes: felt improve consistency of care through better communication and reciprocal learning. Hygiene students reported an improved understanding of patient's complexity and felt it raised their confidence to speak about oral and general health issues.
Wallace, J.P et al. 2017<br><br>Australia<br><br>Study Type: Qualitative	To measure the effect of a orientation DVD used to prepare dental hygiene students for placement at long term care facilities for older adults	Thoughts and feelings	Final year dental hygiene students (n=38), equal split between control and test groups who attended one of 17 care facilities	Both control and test group found care home environment challenging and very different to the usual clinical environment. The test group watched a professionally produced 30 minute DVD with 4 scenarios played by recently graduated hygiene students and actual care home residents before attending, and helped prepare them for the placement compared to the control group and reduced the time it took for them to feel comfortable in that setting.
Wallace, J.P et al. 2014<br><br>Australia	Dental hygiene students' views on a service-learning residential older care placement program	Thoughts and feelings	Final year dental hygiene students (n=22) attended focus groups	Students volunteered to attend a residential care facility for older adults one 4-hour session a week for 12 weeks.<br>During placement underwent formative and summative assessments, delivered oral mouth care and oral hygiene education to patients and staff.<br><br>Students felt overwhelmed and frustrated at not being able to communicate and build rapport with patients with dementia. They found it took a few

Study type: qualitative				weeks to feel more comfortable in the setting. They felt OHI was difficult without designated clinical set up. They felt pre-placement training with realistic scenarios would've been helpful. They felt care staff support was insufficient due to time constraints.
Nitschke I at al. 2009<br><br>Switzerland and Germany<br><br>Study Type: structured questionnaire	To evaluate attitudes towards 2 undergraduate dental gerodontology curriculums	Undergraduate dental student's attitudes towards clinical aspects of 2 gerodontology curriculums	N=67 paired data sets from undergraduate dental students at 2 dental schools	Students at one dental school participated in three clinical activities (in-house gerodontology clinic, extramural acute geriatrics ward, mobile dental service). In the other dental school they visit a long-term care facility on six occasions within 4 years. Both programmes included elements of theoretical teaching.<br>'outstanding' feedback on a 3-day dentalcare deployment with mobiDen (mobile dental care system for long-term care facilities in Zurich), providing treatment and setting up equipment. At the other university there were no facilities to provide dental treatment to residents of long term care facilities and students reported higher feelings of pity and higher levels of mental strain.
De Visschere, L. et al. 2009<br><br>Belgium<br><br>Study type: Questionnaire	Assess impact of undergraduate geriatric dentistry education on attitudes of recently graduated dentists towards institutionalised elderly people	Knowledge about geriatric dentistry and attitudes towards treating institutionalised elderly patients	N= 132 dentists from 6 dental schools who had graduated during 2004-2006	Overall knowledge of ageing poor and attitudes towards treating institutionalised elderly people negative. There was no impact of a dental undergraduate curriculum on knowledge on ageing or attitude towards institutionalised elderly people as perceived by recently graduated dentists.

References outlined in table:

Ahmad B, Landes D, Moffatt S. Dental public health in action: barriers to oral healthcare provision for older people in residential and nursing care homes: a mixed method evaluation and strategy development in County Durham, North East England. Community Dent Health. 2018 Sep;35(3):136–9. doi:10.1922/CDH_4143AHMAD04. PMID: 30102021.

Angst L, Nüesch N, Grandjean ML, Watson S, McKenna GJ, Srinivasan M. Caries management using silver diamine fluoride and providing domiciliary dental care for dependent older adults: a qualitative study of Swiss dentists. Community Dent Oral Epidemiol. 2023 Jun;51(3):469–82. doi:10.1111/cdoe.12774.

Beaton L, Boyle J, Cassie C, Young L, Marshall J. Engaging vocational dental practitioners in care of the dependent elderly: findings from a pilot project. Br Dent J. 2020;228(4):285–8. doi:10.1038/s41415-020-1257-8.

Bots-VantSpijker PC, Bruers JJM, Bots CP, Vanobbergen JNO, De Visschere LMJ, De Baat C, et al. Opinions of dentists on the barriers in providing oral health care to community-dwelling frail older people: a questionnaire survey. Gerodontology. 2016 Jun;33(2):268–74. doi:10.1111/ger.12155.

Caplan DJ, Ghazal TS, Cowen HJ. Effect of Receiving Dental Treatment on mortality among nursing facility residents. Spec Care Dentist. 2022;42(1):3–8. doi: 10.1111/scd.12641. PMID: 34403522.

Castelaz M, Heeren T, Hartshorn JE, Nwachukwu PC, Levy SM, Reynolds JC. Perceptions and experiences with a virtual dental home teledentistry program among community health center and long-term care facility staff: a qualitative study. J Public Health Dent. 2025. doi:10.1111/jphd.12683.

Chowdhry N, Aleksejūnienė J, Wyatt C, Bryant R. Dentists' perceptions of providing care in long-term care facilities. J Can Dent Assoc. 2011;77:b21.
Clark R, Yates C, Howie G. A service evaluation of domiciliary medical emergency equipment and domiciliary medical risk assessment in the West Midlands, UK. J Disabil Oral Health. 2021;22(2):22–30.

De Visschere L, Van Der Putten G-J, De Baat C, Schols J, Vanobbergen J. The impact of undergraduate geriatric dental education on the attitudes of recently graduated dentists towards institutionalised elderly people. Eur J Dent Educ. 2009;13(3):154–61. doi:10.1111/j.1600-0579.2008.00555.x. PMID: 19630934.

Emanuel R, Quach J, Patel P, Witton R, Machuca-Vargas C, Taylor E. The attitudes of dental therapists, dental therapy educators and dental therapy students in the South of England towards domiciliary dentistry: a qualitative study. Br Dent J. 2024 Jan 25;1–5. doi:10.1038/s41415-023-6716-6.

Ericson D, Carlsson P, Gabre P, Wårdh I, Zimmerman M, Sjögren P. Effect of a single application of silver diamine fluoride on root caries after 12 months in institutionalised older adults: a randomised clinical trial. Gerodontology. 2023;40(3):390–7. doi:10.1111/ger.12668.

Fairhall TJ, Thomson WM, Kieser JA, Broughton JR, Cullinan MP, Seymour GJ. Home or away? Differences between home- and clinic-based dental examinations for older people. Gerodontology. 2009;26(3):179–86. doi:10.1111/j.1741-2358.2008.00263.x.

Geddis-Regan AR, O'Connor RC. The impact of age and deprivation on NHS payment claims for domiciliary dental care in England. Community Dent Health. 2018;35(4):223–7. doi:10.1922/CDH_4355Geddis-Regan05.

Geibel AM, Keutel C, Kildal D, M-A G. Mobile dental radiology: evaluation of quality requirements for radiographs applying handheld mobile radiography. Gerodontology. 2025. doi:10.1111/ger.70023.

Hearn L, Slack-Smith L. Engaging dental professionals in residential aged-care facilities: staff perspectives regarding access to oral care. Aust J Prim Health. 2016;22(5):445–51. doi:10.1071/PY15028.

Huynh J, Donnelly LR, Brondani MA. Exploring dental student participation in interdisciplinary care team conferences in long-term care. Gerodontology. 2017;34(2):249–56. doi:10.1111/ger.12259.

Janssens L, Phlypo I, Geddis-Regan A, Petrovic M, Janssens B. Care home managers' perspectives on domiciliary dental care: a qualitative study. BMC Geriatr. 2025;25(1):323. doi:10.1186/s12877-025-06005-5.

Jogezai U, Riches T, Townsend D, Abercrombie C. Introduction of a Nomad Pro handheld dental X-ray unit for radiography in a special care setting. J Disabil Oral Health. 2016;17(2):78–91.

Kandala K, Archer HR, Moss KL, White B, Thomas BR, Wu D, et al. Comparison of initial dental treatment decisions between in-person and asynchronous teledentistry examinations for people with special health care needs. J Am Dent Assoc. 2024;155(8):687–98. doi:10.1016/j.adaj.2024.05.004.

Kerr E, Watson S, McMullan J, Srinivasan M, McKenna GJ. General dentists' attitudes and perceived barriers in providing domiciliary dental care to older adults in long-term care facilities or their homes in Northern Ireland: a descriptive qualitative study. Gerodontology. 2022 Sep;39(3):257–65. doi:10.1111/ger.12565

Kharbot B, Riegel M, Schwendicke F, Paris S, Göstemeier G. Accuracy and reliability of intraoral 3D scans for diagnostic evaluations in nursing home residents. Gerodontology. 2025. doi:10.1111/ger.12817.

Komulainen K, Ylöstalo P, Syrjälä AM, Ruoppi P, Knuuttila M, Sulkava R, et al. Preference for dentist's home visits among older people. Community Dent Oral Epidemiol. 2012 Feb;40(1):89–95. doi:10.1111/j.1600-0528.2011.00631.x.

Lundqvist M, Davidson T, Ordell S, Sjöström O, Zimmerman M, Sjögren P. Health economic analyses of domiciliary dental care and care at fixed clinics for elderly nursing home residents in Sweden. Community Dent Health. 2015;32(1):39–43.

Major N, McQuistan MR, Qian F. Changes in dental students' attitudes about treating underserved populations: a longitudinal study. J Dent Educ. 2016;80(5):517–25.

Makansi N, Rousseau J, Bedos C, Gauthier L, Morissette L, Ducharme I, et al. Domiciliary dentistry clinics: a multiple case study in the province of Quebec, Canada. BMC Health Serv Res. 2021;21(1):972. doi:10.1186/s12913-021-06788-4. PMID: 34526032.

Marino R, Tonmukayakul U, Manton D, Stranieri A, Clarke K. Cost-analysis of teledentistry in residential aged care facilities. J Telemed Telecare. 2016;22(6):326–32. doi:10.1177/1357633X15608991.

Mintz A, Taylor E, Patel P, Emanuel R. Reported barriers to delivering preventative (oral health) advice and domiciliary dental care. J Disabil Oral Health. 2022;23(1):20–6.

Monaghan N, Morgan M. Oral health policy and access to dentistry in care homes. J Disabil Oral Health. 2010;11(2):61–8.

Monaghan NP, Morgan MZ. What proportion of dental care in care homes could be met by direct access to dental therapists or dental hygienists? Br Dent J. 2015 Dec;219(11):531. doi:10.1038/sj.bdj.2015.919.

Morgan MZ, Johnson IG, Hitchings E, Monaghan NP, Karki AJ. Dentist skill and setting to address dental treatment needs of care home residents in Wales. Gerodontology. 2016 Dec;33(4):461–9. doi:10.1111/ger.12185.

Ng BMM, Samaranayaka A, Ting G, Smith M. Acceptability of teledentistry use among residents and staff in aged residential care facilities in the Otago region of New Zealand. Gerodontology. 2024;41(4):555–69. doi:10.1111/ger.12752.

Nitschke I, Reiber T, Sobotta BA. Undergraduate teaching in gerodontology in Leipzig and Zurich--a comparison of different approaches. Gerodontology. 2009;26(3):172–8. doi:10.1111/j.1741-2358.2009.00277.x.

Patel R, Mian M, Robertson C, Pitts NB, Gallagher JE. Crisis in care homes: the dentists don't come. BDJ Open. 2021 Jun 8;7(1):1–6. doi:10.1038/s41405-021-00075-4.

Queyroux A, Saricassapian B, Herzog D, Muller K, Herafa I, Ducoux D, et al. Accuracy of teledentistry for diagnosing dental pathology using direct examination as a gold standard:

results of the Tel-e-dent study of older adults living in nursing homes. J Am Med Dir Assoc. 2017;18(6):528–32. doi:10.1016/j.jamda.2016.12.082.

Shakir H, Marwaha RS, Shah P, Challa S. Effectiveness of silver diamine fluoride in arresting dental caries in residents living in nursing homes: a randomized controlled trial. J Public Health Dent. 2023;83(3):292–8. doi:10.1111/jphd.12578. PMID: 641558018.

Sjögren P, Bäckman N, Sjöström O, Zimmerman M. Patient safety in domiciliary dental care for elderly nursing home residents in Sweden. Community Dent Health. 2015;32(4):216–20.

Smith MB, Thomson WM. 'Not on the radar': dentists' perspectives on the oral health care of dependent older people. Gerodontology. 2017 Mar;34(1):90–100. doi:10.1111/ger.12227.

Szabo KB, Boyd LD, LaSpina LM. Educational preparedness to provide care for older adults in alternative practice settings: perceptions of dental hygiene practitioners. J Dent Hyg. 2018;92(6):16–23.

Tomson M, Watson F, Taylor-Weetman K, Morris AJ, Wilson KI. West Midlands care home dental survey 2011: part 2. Results of clinical survey of care home residents. Br Dent J. 2015;219(7):349–53. doi:10.1038/sj.bdj.2015.758

Turner S, Symeonoglou P, Ross MK. The delivery of dental care to older adults in Scotland: a survey of dental hygienists and therapists. Br Dent J. 2020 Aug 14;1–6. doi:10.1038/s41415-020-1790-5.

Wallace JP, Blinkhorn AS, Blinkhorn FA. A qualitative study examining the preparedness of dental hygiene students for a service-learning placement in residential aged care. Int J Dent Hyg. 2017;15(1):30–6. doi:10.1111/idh.12157.

Wallace JP, Blinkhorn AS, Blinkhorn FA. An assessment of the educational value of service-learning community placements in residential aged care facilities. Int J Dent Hyg. 2014;12(4):298–304. doi:10.1111/idh.12080.

Werbrouck A, Schmidt M, Annemans L, Duyck J, Janssens B, Simoens S, et al. Oral healthcare delivery in institutionalised older people: a health-economic evaluation. Gerodontology. 2022;39(2):107–20. doi:10.1111/ger.12530.

Yoon MN, Compton SM. Building professional competence in dental hygiene students through a community-based practicum. Int J Dent Hyg. 2017;15(4):e119–27. doi:10.1111/idh.12233.

Zaidman B, Awojobi O, Lewis D, Dickinson C, Gallagher JE. Development of a domiciliary dentistry risk assessment form by means of modified e-Delphi study using an expert panel. J Disabil Oral Health. 2020;21(4):80–8.
